# Supplementary material for: Collaborative intelligence in AI: Evaluating the performance of a council of AIs on the USMLE
Source: PLOS Digit Health. 2025 Oct 9;4(10):e0000787. doi: 10.1371/journal.pdig.0000787 (PMC12510544; doi:10.1371/journal.pdig.0000787)
Supplement: S2 File — (ZIP) [file pdig.0000787.s002.zip › S2 Data Raw data files containing unprocessed question inputs and LLM outputs including transcripts of deliberations/STEP3_Question-50-1_Type-MC-NJ.docx]

**Facilitator** **[Round 1]:** A 35-year-old woman comes to the office for follow-up of chronic pelvic pain. The patient has had deep pelvic pain since undergoing total abdominal hysterectomy and radiation therapy for treatment of cervical cancer 5 years ago. She reports constant, severe aching pain; she rates the pain at its maximum as a 10 on a 10‐point scale. The pain worsens during intercourse. All subsequent cancer follow-up has been negative. Medical history otherwise is unremarkable. The patient has taken oxycodone for her pain for 3 years, but she now reports that the oxycodone only partially relieves her pain. She requests either an increased dose of oxycodone or a change in her prescription to controlled-release morphine, which was recently given to her by a friend. She has smoked one pack of cigarettes daily for the past 15 years. She does not drink alcoholic beverages. BMI is 18 kg/m2. She appears lethargic. She is oriented to person, place, and time but speaks slowly with slurred speech. Vital signs are normal. Examination of the skin shows no abnormalities. Pupils are pinpoint and react sluggishly. Urine toxicology screening is positive for diazepam, methadone, and oxycodone. The patient returns to the office 1 week later to discuss these results. Which of the following is the most appropriate opening remark? (A) ""I am concerned that you are abusing pain medicine. I would like for you to consider a substance use disorder treatment program."" (B) ""I cannot be your physician any longer because of your drug use disorder. I'm going to give you 30 days to find a new physician."" (C) ""I would like to know where you have been getting the methadone and diazepam that you are taking."" (D) ""The test showed that you take other drugs that I have not prescribed to you. I must report this to the police.""

**AI_B:** (1) RESTATING THE QUESTION: The patient is a 35-year-old woman who has been experiencing chronic pelvic pain since having a total abdominal hysterectomy and radiation therapy for cervical cancer five years prior. Her pain is severe and she has been using oxycodone for relief over the past three years, however, she reported that it is no longer as effective as it once was. Despite her treatment for cervical cancer being successful, her pain, coupled with the finding of several drugs in her system which she hasn't been prescribed (diazepam and methadone), could suggest substance misuse or addiction. The question asks what the best starting point for conversation might be when discussing these results with her. (2) PLANNING: Several assumptions can be made from these findings. Firstly, one could assume that the patient is self-medicating due to her pain being poorly controlled. However, it is also possible to assume that the patient could be developing a dependency on these substances. While both assumptions are worth considering, the presence of substances not prescribed by the physician in her system seems to indicate that there could be an issue of substance misuse that needs to be prioritized clinically. (3) CLINICAL REASONING: It is necessary to consider that the patient's request for a higher dose or a change of prescription could be a sign of developing tolerance. However, the presence of other substances in her system suggests a more complex picture. Diazepam and methadone are substances often linked with substance misuse and are not typically utilized for managing chronic pain associated with post-radiation and post-hysterectomy changes. The finding would suggest the patient may be using substances to cope with her pain, rather than as a routine treatment regimen. (4) CONCLUDING: Considering these circumstances, it would be crucial to approach the conversation with the patient in a non-confrontational, empathetic manner. The focus should be on ensuring the safety and wellbeing of the patient rather than punitive or accusatory approaches. (5) RESPONSE: Thus, the most appropriate opening remark would be: (A) "I am concerned that you are abusing pain medicine. I would like for you to consider a substance use disorder treatment program."

**AI_D:** RESTATING THE QUESTION: This clinical scenario presents a 35-year-old woman with chronic pelvic pain history since her treatment for cervical cancer 5 years ago. She's been taking oxycodone for pain relief for 3 years but has asked for a higher dose or a switch to controlled-release morphine. Her other symptoms—lethargy, slurred speech, and slow-reacting pinpoint pupils—along with positive urine toxicology screening for diazepam, methadone, and oxycodone suggest the potential misuse of prescription medications. The task involves deciding the most appropriate way to open the discussion about these findings. PLANNING: Taking a non-judgmental, empathetic, and patient-centered approach is crucial in such scenarios. The patient may be trying to self-manage her relentless pain, which would suggest chronic pelvic pain syndrome and/or opioid misuse disorder. Therefore, our assumptions are: 1. The pelvic pain she's experiencing is genuine. 2. Her request for a higher dose or switch in medication is a plea for better pain management. 3. She might be self-medicating with methadone and diazepam, showing possible signs of substance misuse. CLINICAL REASONING: Observations like lethargy, slurred speech, and pinpoint pupils are common symptoms of opioid intoxication. Positive test results for diazepam, methadone, and oxycodone hint towards potential substance misuse/abuse. But, as clinicians, we must approach this with empathy and non-judgment. Jumping to the conclusion of an abuse disorder can lead to loss of trust. To manage this best, we must validate her pain, understand her better, and introduce the topic of potential side-effects or toxicity of her current medication regime. CONCLUDING: Even in the face of evidence for substance misuse, it's essential to navigate this conversation delicately, focusing first on the patient’s perception of her situation and acknowledging her pain experience. We want to reaffirm her autonomy and ability to manage her pain, expressing concerns about the side-effects and potential harm of the current misuse of medications. RESPONSE: Hence, the most appropriate response should be: "I am concerned that you are abusing pain medicine. I would like for you to consider a substance use disorder treatment program" (Choice A). It addresses both her chronic pelvic pain and possible substance misuse in a supportive way without jeopardizing the physician-patient relationship.

**AI_C:** RESTATING THE QUESTION: What's being asked is how best to initiate a sensitive conversation with a 35-year-old woman who presents with chronic pelvic pain following her total abdominal hysterectomy and radiation therapy for cervical cancer. She has been using oxycodone, a very potent opioid, to manage her pain for three years, but is now suggesting either an increase in her dose or a switch to controlled-release morphine. Her lethargic appearance and slow, slurred speech, in conjunction with pinpoint, slow-reacting pupils and a positive toxicology screen for diazepam and methadone, indicate potential opioid misuse or abuse. PLANNING: There are two main assumptions here. First, she needs more efficient analgesics because her pain is not well controlled, hence her request for a dose increase or change to controlled-release morphine. Second, and perhaps more concerning, is the presence of diazepam and methadone, which the physician has not prescribed, and her physical examination findings can suggest opioid abuse that needs to be addressed carefully and effectively. The notion of tapering opioids or a violation of trust by self-medication with other substances should be considered. The important clinical priority here is to address the potential opioid misuse or abuse while maintaining a productive and non-judgmental dialog. CLINICAL REASONING: Radiation and surgery might have caused her chronic pelvic pain, and opioids are often used for pain management for such patients. However, she also presents clinical signs consistent with opioid misuse or abuse, like lethargy, slow speech, pinpoint pupils, and the presence of substances not prescribed in her serum toxicology results, like diazepam and methadone. It raises the question of opioid misuse or abuse, even in the context of chronic pain, that requires intervention. CONCLUDING: In order to ensure the health and safety of the patient, a conversation needs to be initiated concerning the potential opioid misuse and abuse. However, the approach must be empathetic, patient-centered, and non-judgmental. It should focus on concerns about her health and wellbeing. RESPONSE: Thus, the most appropriate opening remark would be option (A) ""I am concerned that you are abusing pain medicine. I would like for you to consider a substance use disorder treatment program."" It embodies empathy and concern for her wellbeing and indicates a path forward that may help her manage her pain more safely and effectively.

**AI_E:** Restating the question: The situation at hand pertains to a 35-year-old woman who is experiencing chronic pelvic pain following a total abdominal hysterectomy and radiation therapy for cervical cancer treatment. Despite using oxycodone for 3 years, she reports partial relief and requests dosages of oxycodone or an alternative prescription to hair controlled-release morphine. Unsettling treatments such as lethargy, slow and slurred speech, and the presence of diazepam and methadone in her urine toxicology suggest possible drug misuse. The question, in this context, requires identifying the most suitable opening statement when she returns for a follow-up visit. Planning: The assumptions, in this case, relate to potential drug dependence or misuse prompted by chronic pelvic pain relief. The sustainability of her current pain management plan involving oxycodone could be reconsidered. Besides a potential increase in dosage or swapping prescriptions, it could be hypothesized that the presence of diazepam and methadone indicate illicit self-medication to enhance pain relief effects. This suggests a need for exploring substance abuse or dependency and planning suitable treatments. Clinical reasoning: Presenting symptoms such as lethargy, slurred speech, and sluggish pupil reactions might add to the alternate assumption of substance misuse. The patient's chronic pain, failure of oxycodone to provide complete relief, and demands for increased dosage or alternative prescription also point towards a potential substance use disorder. A comprehensive approach addressing her pain management needs and potential substance use disorder should be clinically prioritized. Concluding: Analyzing these factors, it appears supportive, and patient-centric dialogue would be the most appropriate opening remark during her next visit. Based on clinical reasoning, it seems essential to address her chronic pain, the insufficient relief from oxycodone, and potential substance misuse symptoms. This would involve discussing available treatment options including, coping strategies for substance abuse, rather than confrontational or threatening remarks. Response: In this regard, the most appropriate opening remark would be option (A) "I am concerned that you are abusing pain medicine. I would like for you to consider a substance use disorder treatment program". This approach opens up an opportunity for a collaborative discussion about her pain management and potential implications of substance abundance. Confrontational, accusative or non-supportive remarks could hinder building a cooperative patient-doctor relationship required for effective treatment planning and may not be effective for addressing the complex interrelated issues of chronic pain and potential substance use disorder seen in this case.

**AI_A:** RESTATING THE QUESTION: This is a scenario about a 35-year-old woman who continues to experience chronic pelvic pain years after a total abdominal hysterectomy and radiation therapy for cervical cancer. She has attained some partial relief from oxycodone but signs indicate increasing physical dependence. Her condition has raised alarm because urine toxicology results indicate the presence of substances not prescribed for her. The concern here is how I, as a healthcare provider, should open a conversation addressing her case in a considerate and clinically appropriate manner. PLANNING: The assumptions underlying this case include that (1) the patient's chronic pelvic pain may still be related to her previous cervical cancer and subsequent treatments; (2) the patient is experiencing analgesic tolerance, and potentially opioid dependence and abuse; (3) the chronic use of opioid medication and the traces of unauthorized substances in her urine screen may indicate a developing or already present substance use disorder; and (4) the slurred speech and physical lethargy could be side effects of her medication or symptoms of polysubstance use. It is crucial to evaluate a more compassionate assumption, that the patient may not fully understand the potential risks and implications of her drug use or is struggling to manage her chronic pain, and therefore requires a sensitive yet comprehensive professional intervention rather than punitive action. CLINICAL REASONING: Addressing these assumptions using my clinical thought process, it is important first to consider the patient's intense and persistent pain after her cervical cancer treatments. This could underscore an increasing dependence on opioid analgesics, as evidenced by her desire to have her current dosage increased or to be switched to a different potent opioid. Furthermore, the presentation of slurred speech and physical lethargy, when combined with the results of her urine toxicology, suggests a possible disorder of substance use. Her BMI also suggests that she is underweight, which may be a physical manifestation of her overall health status, potentially linked to her chronic pain issue as well as her potential substance use. As a clinician, my primary role is to support and guide my patients towards healthier outcomes. To this end, it would be necessary to open a dialogue that recognizes the complexity of her situation and avoids blame or judgment. CONCLUDING: After my careful examination, I determined that it would be crucial to approach her with the intention of understanding and supporting her, rather than immediately suggesting a substance abuse program or terminating the doctor-patient relationship. RESPONSE: Therefore, with all these factors considered, the most appropriate response would be to communicate, "(A) I am concerned that you are abusing pain medicine. I would like for you to consider a substance use disorder treatment program." This remark opens up the conversation about the possibility of a substance use disorder while expressing a genuine concern for her wellbeing and suggesting a potential solution.

**Facilitator** The consensus among the AI responses is that the most appropriate opening remark for the 35-year-old woman who is experiencing chronic pelvic pain and showing signs of potential substance misuse would be: "I am concerned that you are abusing pain medicine. I would like for you to consider a substance use disorder treatment program." This statement, option (A), is recommended as it opens up a conversation about the possibility of a substance use disorder while expressing genuine concern for her wellbeing and suggesting a potential solution. The AI responses agree that the patient's chronic pelvic pain, her request for an increased dose or a change in her prescription, and the presence of diazepam and methadone in her urine toxicology suggest a potential substance use disorder. However, it is also recognized that the patient may be self-medicating due to her pain being poorly controlled. Therefore, it is crucial to approach the conversation with empathy, understanding, and without judgment. The focus should be on ensuring the safety and wellbeing of the patient rather than punitive or accusatory approaches. The conversation should be patient-centered, recognizing the complexity of her situation, and should aim to guide her towards healthier outcomes. It is important to validate her pain, understand her better, and introduce the topic of potential side-effects or toxicity of her current medication regime. In conclusion, the consensus is that the conversation should be initiated in a supportive and non-confrontational manner, focusing on her health and wellbeing, and suggesting a substance use disorder treatment program as a potential solution.
